# Competence. Cooperation. Communications. 10 Years in Medical Assessment Networks – tenth anniversary at Heidelberg

**DOI:** 10.3205/zma000978

**Published:** 2015-10-15

**Authors:** Jana Jünger

**Affiliations:** 1 Medizinische Universitätsklinik Heidelberg, Allgemeine Innere Medizin und Psychosomatik, Heidelberg, Deutschland; 2 Kompetenzzentrum für Prüfungen in der Medizin Baden Württemberg, Heidelberg, Deutschland

## Text

The "Umbrella Consortium for Assessment Network (UCAN)" is celebrating its tenth anniversary on** April 20^th^-22^nd^ 2016**. The Partners Meeting entitled

**Competence. Cooperation. Communications.**

**10 Years in Medical Assessment Networks**

as well as an anniversary celebration will be held at the Schlosshotel Molkenkur, and a pre-conference is scheduled in the Medical Clinic Heidelberg. 

We are looking forward to your contribution and invite you to submit your abstracts on the following topics until **30^th^ November 2015**:

1. Competence-based assessment
2. Quality assurance of medical exams
3. The implementation of the NKLM in assessment networks
4. Innovative assessment formats
5. Programmatic Assessment (Gesamtprüfungsprogramm)
6. Competence-based Progress Test
7. Review of foreign professional qualifications
8. Technical trends in assessment, implementation and evaluation
9. Assessing communicative, professional and inter-professional competencies
10. Discipline-specific cooperations in the field of assessment

For more information please see our conference website www.ucan-assess.org/cms/de/p10.

## Competing interests

The author declares that she has no competing interests.

